# Enteropathogenic infections modulate intestinal serotonin transporter (SERT) function by activating Toll-like receptor 2 (TLR-2) in Crohn’s disease

**DOI:** 10.1038/s41598-021-02050-3

**Published:** 2021-11-19

**Authors:** Ahmad Qasem, Abed Elrahman Naser, Saleh A. Naser

**Affiliations:** 1 Division of Molecular Microbiology, Burnett School of Biomedical Sciences, College of Medicine, University of Central Florida, Orlando, FL USA; 2 Philadelphia College of Osteopathic Medicine, Suwanee, GA USA

**Keywords:** Gastrointestinal diseases, Immunological disorders, Infectious diseases, Immunology, Microbiology, Molecular biology, Physiology, Gastroenterology

## Abstract

Serotonin (5-hydroxytryptamine [5-HT]) is an intestinal neuromodulator that regulates several essential enteric physiological functions such as absorption or secretion of fluids, and peristaltic reflexes. Availability of the intestinal 5-HT is dependent on serotonin transporter (SERT), which uptakes 5-HT and facilitates its degradation. Interestingly, Toll-like receptor 2 (TLR-2) is co-localized with 5-HT, which suggests a possible impact of neuroendocrine cells in the inflammatory response through TLR-2 activation. Serum 5-HT levels were measured in 80 Crohn’s disease (CD) patients and 40 healthy control subjects. Additionally, fully differentiated Caco-2 monolayers were infected with *Mycobacteria paratuberculosis* (MAP), *L. monocytogenes,* or *M. smegmatis* in the presence of exogenous 5-HT at different concentrations. Cells were subsequently harvested and used for measuring SERT activity, RNA isolation followed by RT-PCR, protein quantification, and tissue damage markers (DHE, LDH, GSH and MDA). TLR-2 intracellular signaling pathways were assessed by pre-incubating Caco-2 monolayers with selective blockers of ERK, cAMP/PKA, p38 MAPK, and 5-HT_3_ receptors. MAP-infected CD patients (*N* = 40) had higher serum 5-HT levels (462.95 ± 8.55 ng/mL, *N* = 40) than those without MAP infection (385.33 ± 10.3 ng/mL, *N* = 40). TLR-2 activation by enteropathogenic bacteria inhibited SERT activity in the presence of exogenous 5-HT by up to 50%. These effects were increasing gradually over 72 h, and MAP infection had the greatest effect on SERT inhibition when cells were exposed to 5-HT in a concentration dependent manner. Additionally, inhibition of SERT activity was accompanied with higher levels of pro-inflammatory cytokines (TNF-α, IL-6, IL-8) and oxidative stress markers (DHE, LDH and MDA), whereas SERT expression and protein level were downregulated. Consequently, inhibition of TLR-2 and p38 MAPK signaling or blocking 5-HT_3_ receptors restored SERT activity and reduced the production of pro-inflammatory cytokines, as reflected by the downregulation of oxidative stress and tissue damage markers. The involvement of TLR-2 in the intestinal pathology might be concluded not only from its innate immune role, but also from its effect on modulating the intestinal serotonergic response. Ultimately, regulating the intestinal serotonergic system can be therapeutically exploited to mitigate other enteropathogenic infections, which will help in understanding the gut-microbiome-brain connection.

## Introduction

Serotonin or 5-hydroxytryptamine (5-HT) is a monoamine neurotransmitter that plays a crucial role in the central nervous system by regulating body temperature, sleep pattern, appetite and cognitive functions^1^. However, over 90% of 5-HT is synthesized and stored in enterochromaffin cells of the gastrointestinal (GI) tract^2^. When the intestinal 5-HT interacts with its receptors, it regulates several GI physiological functions such as absorption or secretion of fluids and peristaltic reflexes^3^. There are seven major subtypes of 5-HT receptors (5-HT_1_ to 5-HT_7_), and 5-HT_3_ receptors are the only ligand-gated ion channel, while the other 5-HT receptor subtypes are G-protein coupled receptors^4^. Expression pattern of 5-HT_3_ in the intestinal mucosa mediates a variety of GI processes, whereas it also contributes to development of IBS and gastroesophageal reflux disease (GERD)^5^. Therefore, 5-HT_3_ antagonists (setrons) have been widely used to treat or prevent nausea and vomiting, and this class of drugs has been effectively used to mitigate symptoms of irritable bowel syndrome (IBS)^6^. In acetic-colitis animal model, several 5-HT_3_ antagonists showed anti-inflammatory effects by attenuating the production of IL-1, IL-6 and TNFα^7^. Although these data are compelling, the mechanism by which 5-HT_3_ antagonists exerts its anti-inflammatory effects remains unclear.

Numerous studies have reported that 5-HT_3_ receptors are expressed in immune cells including macrophages, dendritic cells and T cells^8,9^. These findings support the role of 5-HT_3_ receptors in inflammatory and immune responses^8,9^. More importantly, a recent clinical study has shown that serum 5-HT is significantly elevated among Crohn’s disease (CD) patients and it can be used to differentiate between active and refractory disease state, which emphasizes on the potential role of 5-HT in diagnosing active CD and its auxiliary indication for disease remission^10^. Likewise, higher levels of 5-HT were found in the intestinal tissue of inflammatory bowel disease (IBD) patients when compared to healthy controls^11^. These findings suggest that 5-HT may be an integral component of the GI inflammatory process^11^.

The function of 5-HT is terminated by sodium dependent serotonin transporter (SERT), which rapidly uptakes 5-HT and facilitates its degradation^3^. This monoamine transporter protein is encoded by *SLC6A4* gene, and serves as a primary drug target for antidepressant medications^12^. Impaired SERT function is involved in the development of diarrheal conditions, whereas SERT hyperactivity promotes constipation^13^. Therefore, SERT has been considered a critical regulator of 5-HT availability and serotonergic neurotransmission in the GI tract^14^.

Interestingly, Toll-like receptors (TLRs) may be involved in the regulation of intestinal epithelium neuroendocrine activity^15^. Specifically, TLR-1, TLR-2 and TLR-6 are co-localized with 5-HT, which suggests a possible impact of neuroendocrine cells in immune response by TLR activation^16^. Recent study have shown that ligand activation of TLR-2 inhibits SERT activity, but the interaction of TLR-2 and the serotonergic system requires further investigation. In addition, excess level of 5-HT was found to enhance virulence of *Pseudomonas aeruginosa* by activating bacterial quorum sensing^17^. Since IBD has been strongly linked to microbial association such as *Mycobacterium avium paratuberculosis* (MAP) and *Listeria monocytogenes*^18–20^, we were intrigued to investigate the role of enteropathogenic infections in altering SERT activity through TLR2 activation.

The aim of this work is to provide an insight toward understanding how pathogenic bacteria induce inflammatory response by increasing an excess level of 5-HT, and to elucidate a potential role of 5-HT_3_ receptor antagonists in suppressing the intestinal inflammation in IBD. Ultimately, regulating serotonin levels in the GI tract can be therapeutically exploited to mitigate other enteropathogenic infections, which will help in understanding the gut-microbiome-brain connection.

## Results

### Higher serum 5-HT level in MAP-infected CD patients compared to CD patients without MAP infection or healthy controls

**Figure 1:**
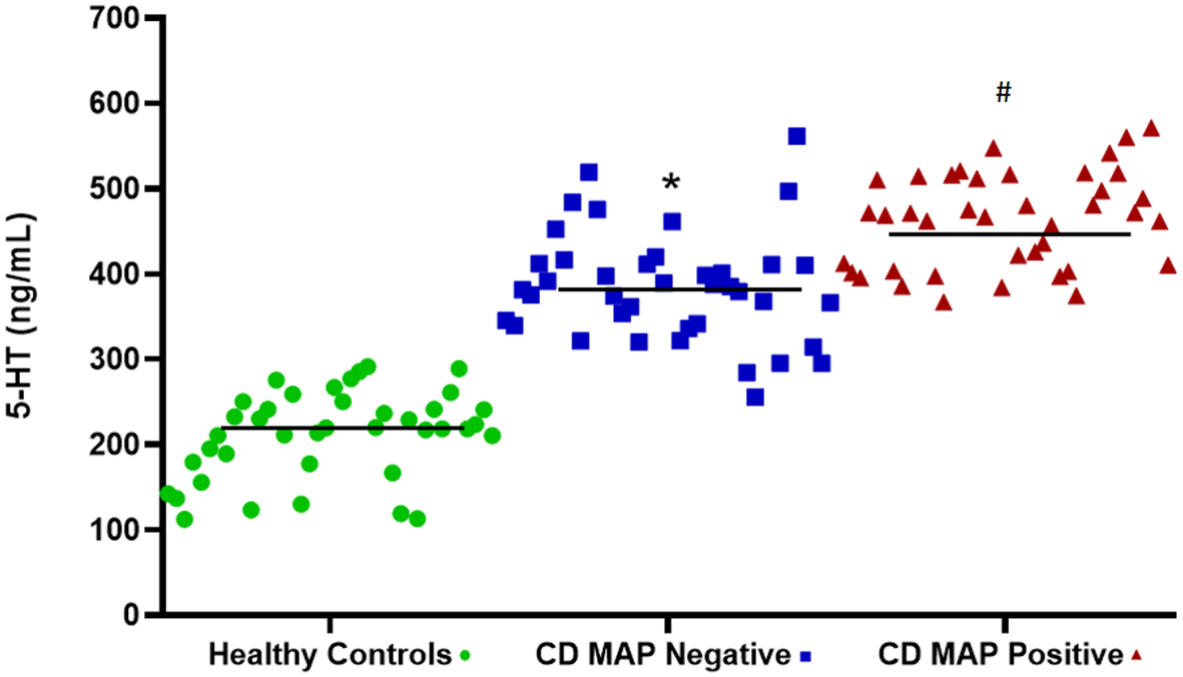
Clinical serum 5-HT levels determined by serotonin ELISA assay (Eagle Biosciences, Amherst, NH) in CD patients with or without MAP infection vs healthy control Subjects (*N* = 40 per each group). Values were pre-tested for normal distribution using the Kolmogorov–Smirnov normality test. Significance among experiments was assessed by two-way analysis of variance (ANOVA) followed by Bonferroni correction test. *P value < 0.05 compared to healthy controls. ^#^*P* value < 0.05 compared to healthy controls or CD MAP negative patients.

We measured 5-HT serum level in 40 healthy control subjects, 40 MAP positive CD patients and 40 MAP negative CD patients. Our data indicate a significant higher serum level of 5-HT in CD patients (*N* = 80) in comparison to healthy controls (*N* = 40) [424.14 ± 9.42 ng/mL vs. 211.69 ± 8.11 ng/mL, respectively]. Moreover, CD patients who were MAP infected had a significantly higher serum level of 5-HT (462.95 ± 8.55 ng/mL), when compared to MAP negative CD patients (385.33 ± 10.3 ng/mL) (Fig. 1).

### MAP infection has the highest effect on inhibiting SERT activity in the presence of exogenous 5-HT

**Figure 2:**
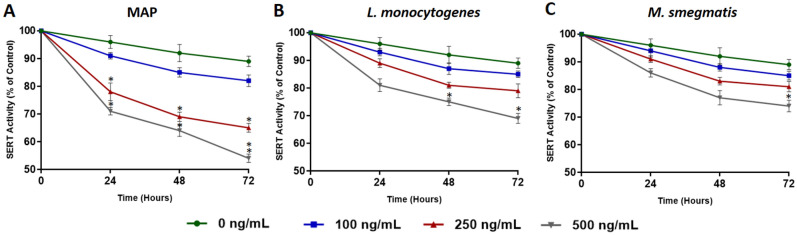
Effects of MAP (**A**), *L. monocytogenes* (**B**) and *M. smegmatis* (**C**) infections on SERT activity in fully differentiated Caco-2 monolayers in the presence of different levels of 5-HT (0, 100, 250, and 500 ng/mL) over 24, 48 and 72 h. Values are presented as percentage activity of control group without infection or 5-HT treatment. Values were pre-tested for normal distribution using the Kolmogorov–Smirnov normality test. Significance among experiments was assessed by repeated measures analysis of variance (ANOVA) followed by Bonferroni correction test. All SERT activity experiments were performed in triplicates using the homogeneous neurotransmitter transporter uptake assay (Molecular Devices Corporation, San Jose, CA). Data are presented as Mean ± SD. *P value < 0.05.

To test the direct effect of bacterial infection on SERT activity, fully differentiated Caco-2 monolayers were infected with MAP, *L. monocytogenes* and *M. smegmatis* in the presence of different levels of 5-HT (0, 100, 250, 500 ng/mL). SERT activity was measured at three time points every 24 h and values were normalized to control group without infection or 5-HT exposure. We noticed that MAP infection had the greatest significant effect on reducing SERT activity when cells were exposed to 250 ng/mL (78.3 ± 3.2%, 69.5 ± 1.7% and 65.2 ± 1.4% at 24, 48 and 72 h, respectively) and 500 ng/mL (71.4 ± 1.5%, 64.3 ± 2.1% and 54.7 ± 1.5% at 24, 48 and 72 h, respectively) of 5-HT (Fig. 2A). Caco-2 monolayers infected with *L. monocytogenes* showed significant reduction of SERT activity when cells were exposed to 500 ng/mL for 48 and 72 h (75.3 ± 1.6% and 69.8 ± 1.8% at 48 and 72 h, respectively) (Fig. 2B). However, *M. smegmatis* infection did not have a significant effect on SERT activity (Fig. 2C).

### MAP infection inhibits SERT expression and protein level through activation of TLR-2 in the presence of exogenous 5-HT

**Figure 3:**
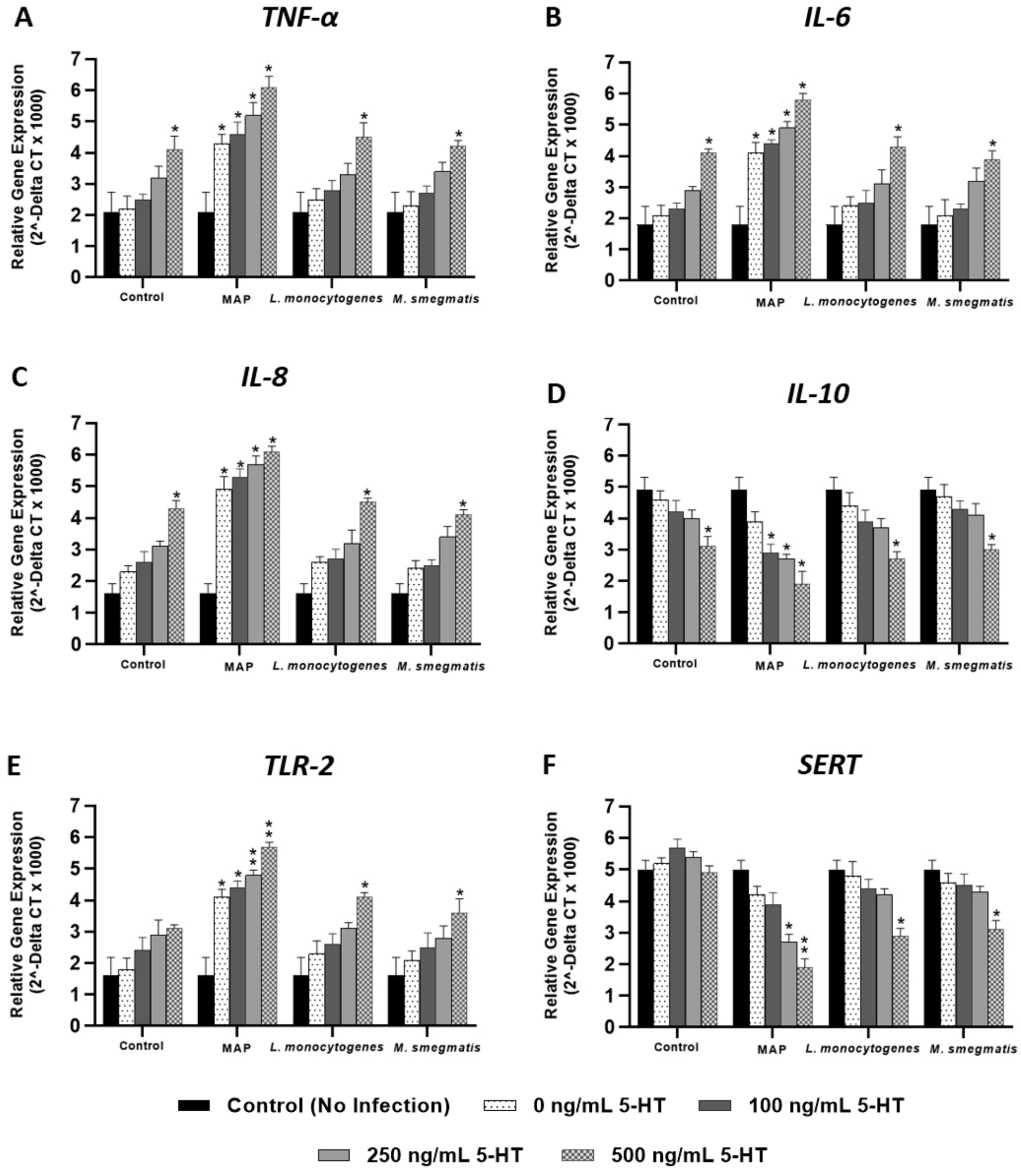
Effects of enteropathogenic bacterial infections on expression of *TNF-α* (**A**), *IL-6* (**B**), *IL-8* (**C**), *IL-10* (**D**), *TLR-2* (**E**) and *SERT* (**F**) in Caco-2 monolayers exposed to exogenous 5-HT levels (0, 100, 250 and 500 ng/mL) for 24 h. Values were pre-tested for normal distribution using the Kolmogorov–Smirnov normality test. Significance among experiments was assessed by two-way analysis of variance (ANOVA) followed by Bonferroni correction test. All RT-PCR experiments were performed in triplicates. Data are presented as Mean ± SD. *P value < 0.05. **P value < 0.001.

**Figure 4:**
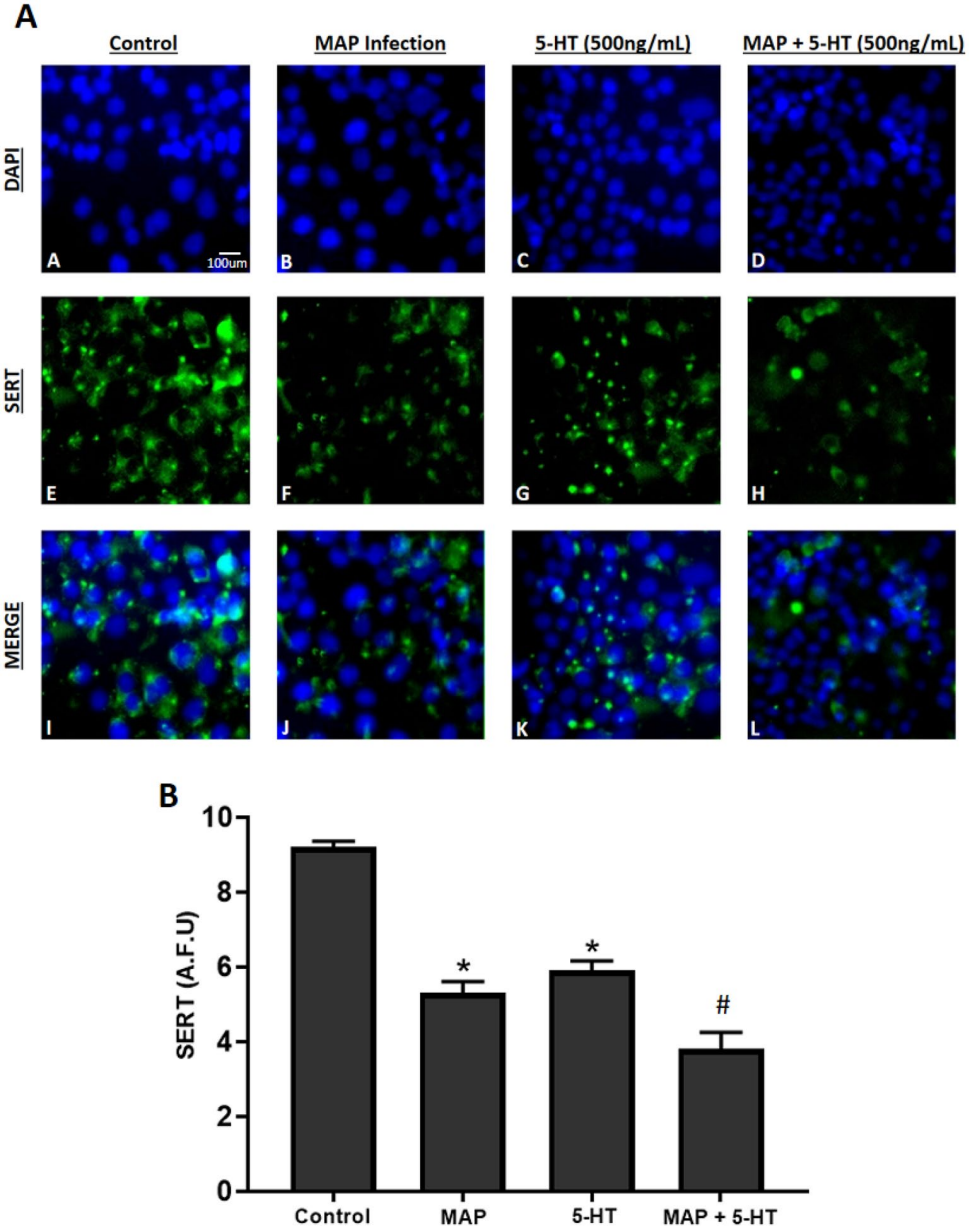
(**A**) Effect of MAP infection on SERT protein level in Caco-2 monolayers in the presence of excess level (500 ng/mL) of 5-HT following 24 h of incubation. Total nuclei are stained with DAPI in blue (**A**–**D**). SERT positive cells are stained in green (**E**–**H**), and merged cells are presented in pink (**I**–**L**). (**B**) Quantitative corrected SERT fluorescence integrated density from control and treated groups. Values were pre-tested for normal distribution using the Kolmogorov–Smirnov normality test. Significance among experiments was assessed by two-way analysis of variance (ANOVA) followed by Bonferroni correction test. All SERT fluorescence staining experiments were performed in triplicates. Data are presented as Mean ± SD. *P value < 0.05 compared to no treatment control. ^#^*P* value < 0.05 compared to no treatment control, MAP, and 5-HT values.

Relative gene expression and protein level of pro-inflammatory cytokines (*TNF-α*, *IL-6*, *IL-8*), *IL-10*, *TLR-2* and *SERT* were measured in fully differentiated Caco-2 monolayers following 24 h of bacterial infection in the presence of different levels of 5-HT (0, 100, 250, 500 ng/mL). Our results indicate that exposure to 5-HT in all tested bacterial infections upregulates *TLR-2* and pro-inflammatory cytokines expression and protein level in a concentration dependent manner (Fig. 3). Consequently, stimulating inflammation resulted in downregulation of *SERT* and *IL-10* expression. These results were significant only in the presence of 5-HT at 500 ng/mL in all groups including cells exposed to 5-HT without bacterial infection, whereas MAP infection induced inflammation and downregulated *SERT* significantly at 100, 250 and 500 ng/mL of 5-HT treatment (Table 1). Specifically, in Caco-2 monolayers exposed to 500 ng/mL of 5-HT, expression and protein level of *TNF-α*, *IL-6* and *IL-8* were about 3 folds higher in MAP-infected monolayers compared to control cells without infection or 5-HT treatment. In contrast, expression and protein level of *SERT* and *IL-10* were progressively decreasing significantly following MAP infection in monolayers exposed to 5-HT at 100, 250 and 500 ng/mL. Since MAP infection caused the highest level of inflammation and inhibition of *SERT* expression in Caco-2 monolayers exposed to an excess level of 5-HT, we further performed fluorescence staining assay for SERT detection in the presence of 5-HT at 500 ng/mL following 24 h of MAP infection. Similarly, 5-HT exposure in MAP infected monolayers showed the highest effect on reducing SERT fluorescence in comparison to MAP infection alone or to 5-HT without MAP infection, which indicates that 5-HT further reduces SERT localization caused by MAP infection (Fig. 4).

**Table 1:** Effects of enteropathogenic bacterial infections on protein level of TNF-α, IL-6, IL-10, IL-8, TLR-2 and SERT in Caco-2 monolayers exposed to exogenous 5-HT levels (0, 100, 250 and 500 ng/mL) for 24 h.

Bacterial infection	5-HT concentration (ng/mL)	TNF-α ± SD (pg/mL)	IL-6 ± SD (pg/mL)	IL-8 ± SD (pg/mL)	IL-10 ± SD (pg/mL)	TLR-2 ± SD (ng/mL)	SERT ± SD (pg/mL)
No treatment No infection	0	63.7 ± 3.2	61.8 ± 5.1	59.3 ± 4.5	117.2 ± 7.1	8.4 ± 1.2	28.3 ± 2.1
	100	78.1 ± 4.3	73.4 ± 1.3	70.4 ± 3.9	107.1 ± 4.3	9.1 ± 2.0	25.2 ± 2.7
	250	89.9 ± 2.9	85.7 ± 2.9	84.7 ± 5.4	92.3 ± 1.9	9.7 ± 1.9	24.5 ± 2.1
	500	172.8 ± 6.3*	163.9 ± 4.8*	169.5 ± 2.8*	71.9 ± 4.7*	11.4 ± 1.4	22.7 ± 3.5
MAP	0	120.1 ± 8.6*	112.6 ± 4.6*	122.4 ± 7.3*	91.6 ± 3.1	14.5 ± 1.2*	23.9 ± 3.1
	100	137.9 ± 4.3*	129.3 ± 2.1*	141.7 ± 5.1*	73.1 ± 5.7*	16.3 ± 1.4*	21.7 ± 1.6
	250	151.3 ± 2.9*	145.4 ± 3.87*	156.2 ± 6.4*	60.3 ± 2.4*	19.6 ± 2.3*	12.2 ± 3.1*
	500	194.8 ± 6.3*	185.2 ± 1.8*	198.3 ± 1.9*	94.3 ± 3.3*	22.5 ± 2.7*	9.6 ± 2.6*
*L. monocytogenes*	0	70.01 ± 6.3	67.3 ± 1.2	74.3 ± 2.5	109.2 ± 5.2	10.1 ± 1.7	26.8 ± 2.6
	100	81.7 ± 5.1	75.1 ± 7.4	86.1 ± 7.4	97.3 ± 6.1	12.6 ± 1.5	25.4 ± 2.3
	250	94.6 ± 3.9	89.6 ± 2.3	97.4 ± 8.6	85.4 ± 1.5	13.2 ± 1.1	21.1 ± 1.7
	500	183.4 ± 4.8*	168.5 ± 3.7*	186.9 ± 6.2*	65.2 ± 2.9*	16.3 ± 2.8*	16.7 ± 3.0*
*M. smegmatis*	0	68.3 ± 2.5	64.2 ± 6.2	70.3 ± 7.3	113.1 ± 5.2	9.3 ± 1.4	27.7 ± 1.3
	100	78.4 ± 4.2	70.1 ± 1.9	81.2 ± 2.7	102.0 ± 6.7	11.2 ± 1.2	25.9 ± 2.6
	250	85.0 ± 3.7	82.7 ± 3.5	88.7 ± 4.2	91.4 ± 2.9	12.7 ± 1.7	23.5 ± 2.1
	500	175.6 ± 5.2*	165.7 ± 4.1*	179.5 ± 3.1*	66.3 ± 3.8*	15.4 ± 1.9*	18.6 ± 2.2*

Values were pre-tested for normal distribution using the Kolmogorov–Smirnov normality test. Significance among experiments was assessed by two-way analysis of variance (ANOVA) followed by Bonferroni correction test. All ELISA experiments were performed in triplicates. *P value < 0.05.

### MAP infection induces the highest level of oxidative stress and tissue damage in Caco-2 monolayers in the presence of exogenous 5-HT

**Figure 5:**
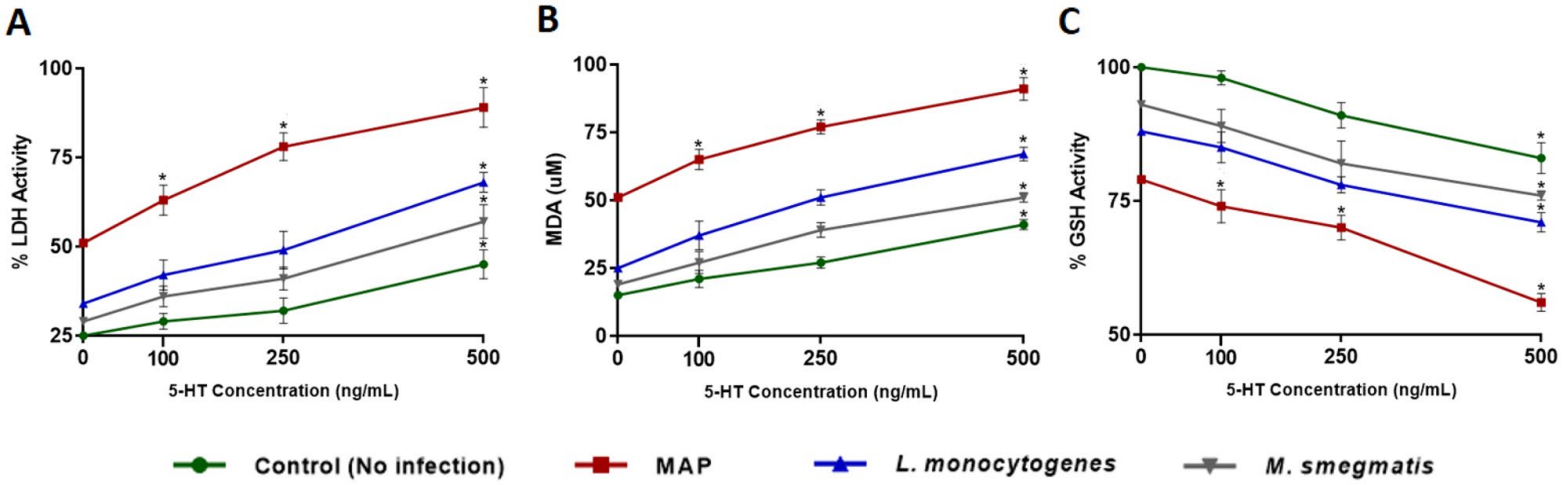
Effects of MAP, *L. monocytogenes* and *M. smegmatis* following 24 h of infection on LDH Activity (**A**) GSH Activity (**B**) and MDA level (**C**) in fully differentiated Caco-2 monolayers in the presence of different levels of 5-HT (0, 100, 250, and 500 ng/mL) following 24 h of incubation. LDH and GSH activity values are presented as percentage activity of control group without infection or 5-HT treatment. Values were pre-tested for normal distribution using the Kolmogorov–Smirnov normality test. Significance among experiments was assessed by repeated measures analysis of variance (ANOVA) followed by Bonferroni correction test. All LDH, GSH and MDA activity experiments were performed in triplicates. Data are presented as Mean ± SD. *P value < 0.05.

**Figure 6:**
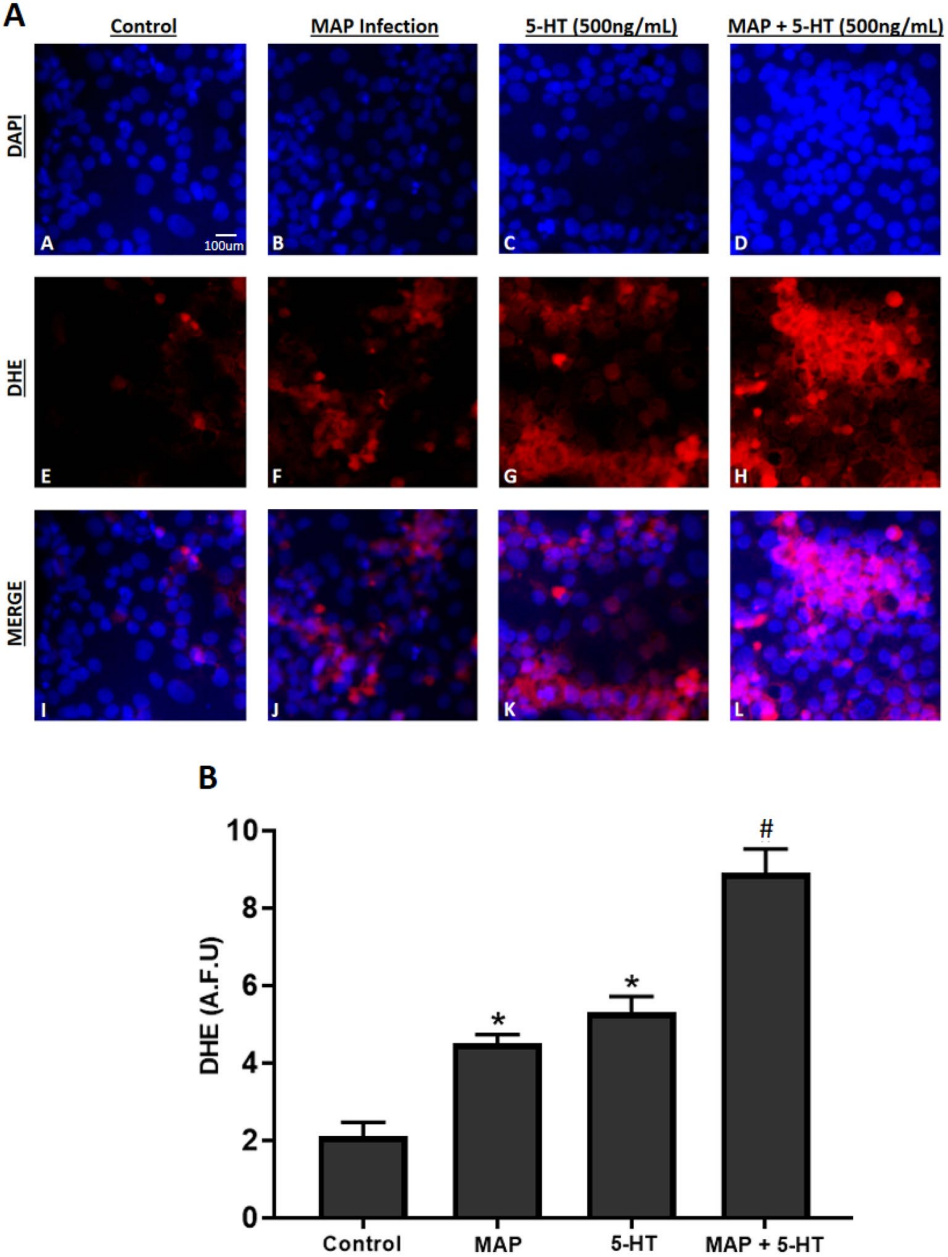
(**A**) Effect of MAP infection on oxidative stress level in Caco-2 monolayers in the presence of excess level (500 ng/mL) of 5-HT following 24 h of incubation. Total nuclei are stained with DAPI in blue (**A**–**D**). DHE positive cells are stained in red (**E**–**H**), and merged cells are presented in pink (**I**–**L**). (**B**) Quantitative corrected DHE fluorescence integrated density from control and treated groups. Values were pre-tested for normal distribution using the Kolmogorov–Smirnov normality test. Significance among experiments was assessed by two-way analysis of variance (ANOVA) followed by Bonferroni correction test. All DHE fluorescence staining experiments were performed in triplicates. Data are presented as Mean ± SD. *P value < 0.05 compared to no treatment control. ^#^*P* value < 0.05 compared to no treatment control, MAP, and 5-HT values.

To further confirm our findings, we assessed the effect of MAP, *L. monocytogenes* and *M. smegmatis* infections on tissue damage and oxidative stress activity by measuring LDH activity, GSH activity and MDA level in Caco-2 monolayers following 24 h of bacterial infection in the presence of different levels of 5-HT (0, 100, 250, 500 ng/mL) and values were normalized to control group without infection or 5-HT exposure. MAP infection showed the highest levels of LDH (51.4 ± 2.1%, 63.7 ± 4.2%, 78.1 ± 3.9% and 89.3 ± 5.6% at 0, 100, 250 and 500 ng/mL, respectively) (Fig. 5A) and MDA (51.2 ± 1.6, 65.8 ± 3.8, 77.2 ± 2.6 and 91.6 ± 4.2 uM at 0, 100, 250 and 500 ng/mL, respectively) (Fig. 5B). In contrast, GSH activity was the lowest in MAP infected caco-2 monolayers (79.1 ± 1.8%, 74.3 ± 3.1%, 70.6 ± 2.3% and 56.3 ± 1.7% at 0, 100, 250 and 500 ng/mL, respectively) compared to all tested groups (Fig. 5C). Addition of 5-HT to the bacterial infection progressively augmented LDH and MDA levels while decreasing GSH activity in a concentration dependent manner, and the result was significant in all tested groups at 500 ng/mL. Since MAP infection caused the highest level of oxidative stress in Caco-2 monolayers exposed to an excess level of 5-HT, we further performed DHE fluorescence staining assay in the presence of 5-HT at 500 ng/mL. Similarly, 5-HT exposure in MAP infected monolayers showed the highest level of DHE fluorescence in comparison to MAP infection alone or to 5-HT without MAP infection, which indicates that 5-HT increases oxidative stress level caused by MAP infection (Fig. 6).

### Inhibition of 5-HT_3_ receptors or p38 MAPK pathway reduce inflammation and oxidative stress by restoring SERT activity in MAP-infected Caco-2 monolayers

**Figure 7:**
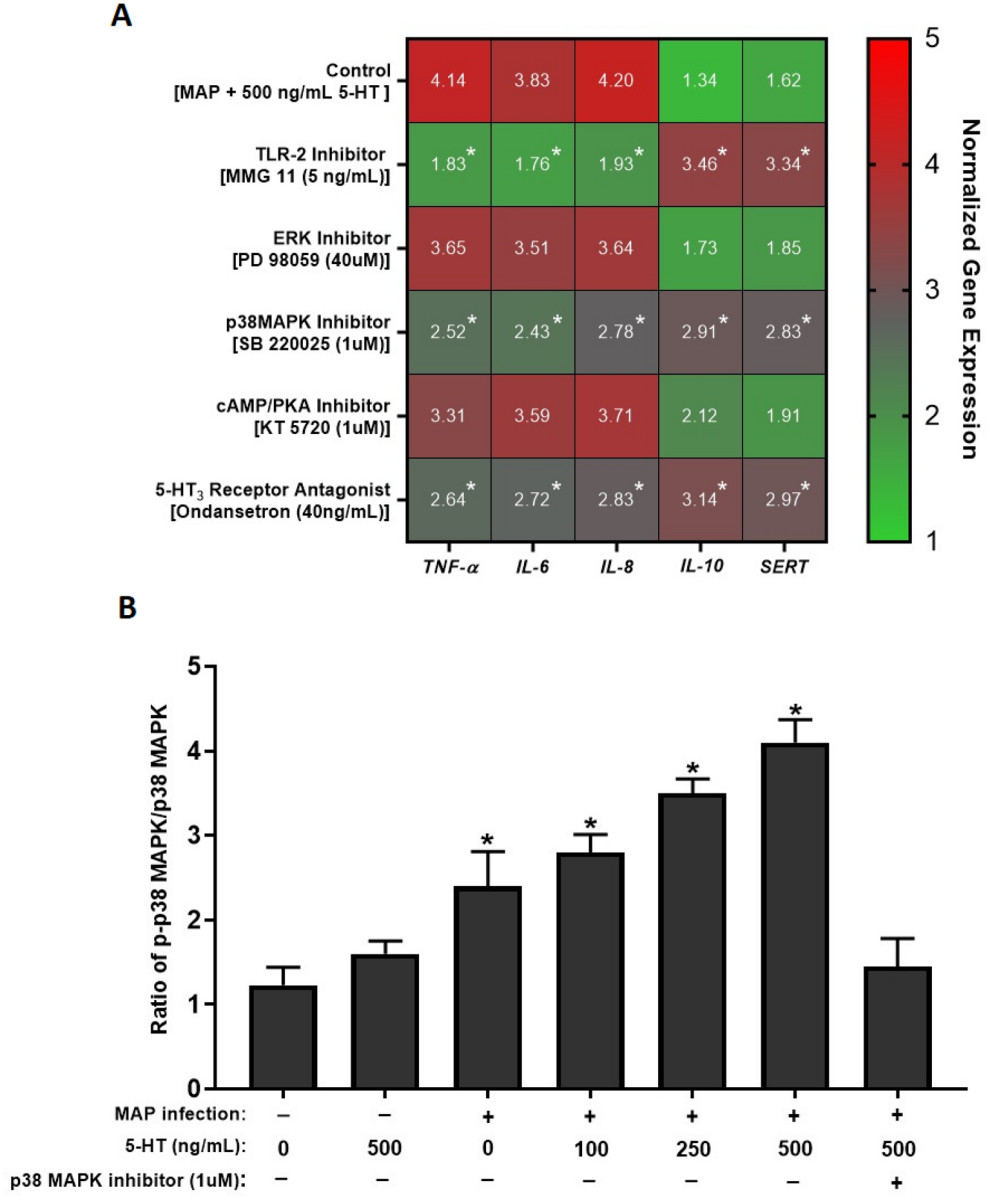
(**A**) Effects of several inhibitors involved in TLR-2 signaling pathway on expression of *TNF-α, IL-6, IL-8, IL-10* and *SERT* in MAP-infected Caco-2 monolayers in the presence of excess level of 5-HT (500 ng/mL) following 24 h of incubation. Values were normalized to relative gene expression level of control monolayers without 5-HT treatment or infection. (**B**) The effect of MAP infection on the ratio of p-p38 MAPK/p38 MAPK in the presence of different levels of 5-HT (0, 100, 250, 500 ng/mL) with or without 1 uM of p38 MAPK inhibitor (SB220025) following 24 h of incubation. Values were pre-tested for normal distribution using the Kolmogorov–Smirnov normality test. Significance among experiments was assessed by two-way analysis of variance (ANOVA) followed by Bonferroni correction test. All RT-PCR and phosphorylation activity of p38 MAPK experiments were performed in triplicates. Data are presented as Mean ± SD. *P value < 0.05.

In order to further analyze the effects of TLR-2 activation on modulating SERT activity and inflammation, we used specific inhibitors of intracellular pathways involved in TLR-2 signaling, including TLR-2 inhibitor [MMG 11 (5 ug/mL)], ERK inhibitor [PD 98059 (40 uM)], p38 MAPK inhibitor [SB 220025 (1 uM)] and cAMP/PKA inhibitor [KT 5720 (1 uM)]. Additionally, we tested the anti-inflammatory effects of 5-HT_3_ receptor antagonist [ondansetron (40 ng/mL)] to show that excess 5-HT is required for sustained inflammatory process caused by MAP infection. In comparison to MAP-infected Caco-2 monolayers exposed to 500 ng/mL 5-HT for 24 h, inhibition of TLR-2 lead to the highest significant decrease in *TNF-α*, *IL-6* and *IL-8* (by 2.26, 2.18 and 2.17 folds, respectively), while it significantly restored expression of *IL-10* and *SERT* (2.58 and 2.06 folds, respectively) (Fig. 7A).

Similarly, inhibition of p38 MAPK and blocking 5-HT_3_ receptors by ondansetron had a significant effect on downregulating the expression of *TNF-α*, *IL-6* and *IL-8* by nearly 2 folds, while these treatments significantly restored expression of *IL-10* and *SERT* (Fig. 7A). In contrast, inhibition of ERK and cAMP/PKA pathways did not have a significant effect on modulating cytokines or *SERT* expression. The results of modulating gene expression were correspondent with the quantified protein level (Table 2). As shown in Table 3, significant reduction in LDH activity and MDA level, in addition to higher GSH and SERT activities were noticed following inhibition of TLR-2 and p38 MAPK or by blocking 5-HT_3_ receptors using physiological level (40 ng/mL) of ondansetron.

**Table 2:** Effects of several inhibitors involved in TLR-2 signaling pathway on protein level of TNF-α, IL-6, IL-8, IL-10 and SERT in MAP-infected Caco-2 monolayers in the presence of excess level (500 ng/mL) of 5-HT following 24 h of incubation.

Treatment	TNF-α ± SD (pg/mL)	IL-6 ± SD (pg/mL)	IL-8 ± SD (pg/mL)	IL-10 ± SD (pg/mL)	SERT ± SD (pg/mL)
Control (MAP + 500 ng/mL 5-HT)	194.8 ± 6.3	185.2 ± 1.8	198.3 ± 1.9	94.3 ± 3.3	9.6 ± 2.6
TLR-2 Inhibitor (MMG 11 (5 ng/mL))	82.3 ± 5.1*	84.1 ± 1.3*	81.9 ± 2.4*	87.8 ± 1.4*	22.2 ± 1.5*
ERK Inhibitor (PD 98059 (40 uM))	169.5 ± 3.7	172.4 ± 5.2	165.5 ± 6.8	61.4 ± 2.7	13.4 ± 2.4
p38 MAPK Inhibitor (SB 220025 (1 uM))	101.2 ± 4.8*	104.7 ± 7.9*	97.1 ± 3.1*	79.3 ± 1.6*	20.1 ± 2.7*
cAMP/PKA Inhibitor (KT 5720 (1 uM))	161.9 ± 2.4	167.6 ± 1.4	158.6 ± 4.2	65.7 ± 3.9	14.4 ± 1.2
5-HT_3_ Receptor Antagonist (Ondansetron (40 ng/mL))	109.7 ± 6.1*	112.1 ± 3.8*	105.3 ± 1.9*	75.1 ± 2.3*	19.3 ± 2.6*

Values were pre-tested for normal distribution using the Kolmogorov–Smirnov normality test. Significance among experiments was assessed by two-way analysis of variance (ANOVA) followed by Bonferroni correction test. All ELISA experiments were performed in triplicates. *P value < 0.05.

**Table 3:** Effects of several inhibitors involved in TLR-2 signaling pathway on the activities of SERT, LDH, GSH and MDA level in MAP-infected Caco-2 monolayers in the presence of excess level (500 ng/mL) of 5-HT following 24 h of incubation.

Treatment	SERT activity ± SD (% of control)	LDH activity ± SD (% of control)	GSH activity ± SD (% of control)	MDA level ± SD (uM)
Control (MAP + 500 ng/mL 5-HT)	71.2 ± 1.5	89.7 ± 5.6	56 ± 1.7	91 ± 4.2
TLR-2 Inhibitor (MMG 11 (5 ng/mL))	88.4 ± 2.7*	39.2 ± 2.3*	86.5 ± 3.4*	34.1 ± 5.2*
ERK Inhibitor (PD 98059 (40 uM))	74.5 ± 1.4	79.1 ± 5.7	64.1 ± 1.7	79.2 ± 3.6
p38 MAPK Inhibitor (SB 220025 (1 uM))	83.7 ± 1.9*	47.4 ± 4.6*	81.7 ± 2.6*	39.4 ± 4.3*
cAMP/PKA Inhibitor (KT 5720 (1 uM))	76.1 ± 2.7	77.3 ± 1.9	68.9 ± 5.1	75.8 ± 1.7
5-HT_3_ Receptor Antagonist (Ondansetron (40 ng/mL))	81.4 ± 3.6*	48.6 ± 2.8*	79.3 ± 3.8*	43.3 ± 2.9*

Values were pre-tested for normal distribution using the Kolmogorov–Smirnov normality test. Significance among experiments was assessed by two-way analysis of variance (ANOVA) followed by Bonferroni correction test. All SERT, LDH, GSH, and MDA activity experiments were performed in triplicates. *P value < 0.05.

Moreover, since the phosphorylated form (p-p38 MAPK) mediates p38 MAPK activity, we measured the ratio of p-p38 MAPK/p38 MAPK pathway following MAP infection in the presence of different levels of 5-HT (0, 100, 250, 500 ng/mL). The results obtained have suggested that MAP infection without 5-HT treatment increases the ratio of p-p38 MAPK/p38 MAPK by about two folds, while 5-HT progressively increases phosphorylation of p38 MAPK following MAP infection in a concentration dependent manner, reaching about four folds higher when cells were exposed to 500 ng/mL (Fig. 7B).

## Discussion

The intestinal mucosal barrier selectively absorbs essential nutrients while preventing the entry of harmful toxins and pathobionts^21^. Moreover, the intestinal epithelium participates in an active immune response by recognizing specific microorganism-associated molecular patterns (MAMPs) through TLRs, which carefully distinguishes commensal from pathogenic bacteria^22^. In this context, TLR-2 plays an integral role in intestinal homeostasis, by recognizing Gram-positive bacteria and mycobacterial lipopeptide^23^. Heterodimeric expression of TLR1 or TLR6 is required for TLR-2 activation, which generates a signal to tolerate or eradicate bacteria, depending on its pathogenicity^22^. Therefore, dysregulation in TLR-2 activity has been implicated in several GI conditions such as IBD and IBS^24–28^.

Recent studies have demonstrated that TLR-2 signaling pathway regulates the intestinal serotonergic system^29^. The presence of 5-HT in the GI tract plays a major role in gut motility, neuronal reflexes and fluid secretion. A compelling evidence has suggested that 5-HT is involved in CD pathogenesis, which is characterized by the prevalence of enterochromaffin cells, higher 5-HT mucosal content, increased activity of tryptophan hydroxylase I (TPH-1), and more importantly, lower expression of SERT, a critical regulator of the intestinal 5-HT availability^10^. In the present study, we have confirmed that serum 5-HT level is significantly higher among CD patients in comparison to healthy subjects, and MAP-infected CD patients had a higher level of 5-HT than those without MAP infection.

We demonstrated that TLR-2 activation in fully differentiated Caco-2 monolayers by enteropathogenic bacteria (MAP, *L. monocytogenes* and *M. smegmatis*) inhibits SERT activity in the presence of exogenous 5-HT. These effects increased gradually over 72 h, and MAP infection had the greatest effect on SERT inhibition when cells were exposed to 5-HT in a concentration dependent manner. Additionally, inhibition of SERT activity was accompanied with higher level of pro-inflammatory cytokines (TNF-α, IL-6, IL-8) and oxidative stress markers (LDH and MDA), whereas SERT expression and protein level were downregulated. These results indicate that TLR-2 activation may repress the intestinal SERT function and availability, leading to excessive accumulation of extracellular 5-HT in the GI tract.

**Figure 8:**
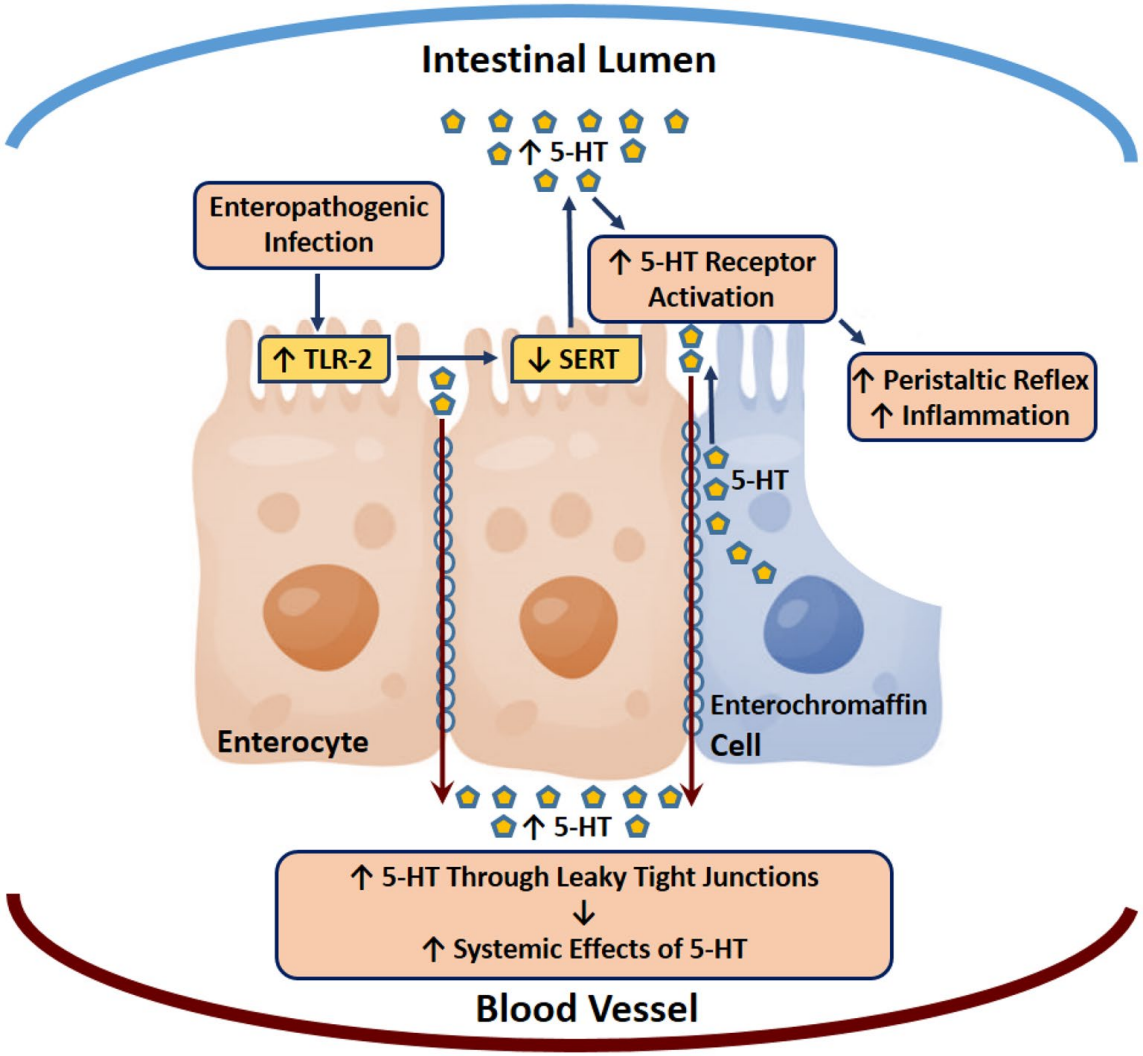
Proposed mechanism for increasing intestinal and serum 5-HT among CD patients. Enterochromaffin cells release 5-HT into the intestinal lumen. Under normal conditions, SERT activity is able to reuptake 5-HT back into the intracellular compartment, which regulates intestinal 5-HT availability. However, enteropathogenic infections cause inflammation by activating TLR-2, which reduces SERT expression and function, leading to accumulation of 5-HT and disruption of the intestinal tight junctions. This results in paracellular movement of 5-HT across the intestinal epithelium to the blood vessels, where it can cause further systemic complications.

Consequently, inhibition of TLR-2 restored SERT activity and reduced the production of pro-inflammatory cytokines, which was reflected by the decrease in oxidative stress and tissue damage markers. Recently, it has been shown that 5-HT accumulation activates colonic nicotinamide adenine dinucleotide phosphate (NADPH) oxidase, which generates reactive oxygen species (ROS)^30^. Furthermore, we previously reported that MAP-infected CD patients had a higher level of glutathione peroxidase (GPx) activity, indicating higher oxidative stress level^31^. Therefore, accumulation of extracellular 5-HT in the presence of MAP infection further induced the pro-inflammatory process and caused higher level of tissue damage. Ultimately, this could affect the intestinal tight junctions that are essential in regulating the physical intestinal barrier, leading to paracellular movement of 5-HT across the intestinal epithelium to the blood vessels, where it can trigger complications in multiple organ-systems (Fig. 8).

In order to investigate the underlying mechanism by which TLR-2 activation inhibits SERT activity, we used specific inhibitors of intracellular pathways involved in TLR-2 signaling. Recent studies have shown that cell signaling associated ERK pathway modulates TLR-2 signaling in macrophages and neuronal cells^32,33^. In addition, the cAMP/PKA pathway has been involved in the TLR-2 immune response^34^. However, we noticed that inhibition of the ERK and cAMP/PKA pathways did not have a significant effect on modulating pro-inflammatory cytokine release or restoring SERT activity. In contrast, inhibition of the p38 MAPK pathway had a significant effect on downregulating inflammation and restoring SERT activity. The results obtained have suggested that 5-HT progressively increases the phosphorylation of MAPK following MAP infection in a concentration dependent manner, which was demonstrated by increasing the ratio of p-p38 MAPK/p38 MAPK. The role of p38 MAPK signaling in modulating SERT function is controversial, since p38 MAPK activation does not have an effect on SERT activity in the brain, but it enhances SERT activity in neuronal cells^35,36^. Our results agree with two previous studies, showing that p38 MAPK activation inhibits SERT activity in the intestinal epithelial tissue^29,37^.

The presence of 5-HT_3_ receptors in the intestinal mucosa mediates a variety of GI processes, including motility and fluids secretion^5^. Therefore, 5-HT_3_ antagonists effectively counteract chemotherapy‐induced emesis^6^. Since 5-HT_3_ receptors are expressed in macrophages and T-cells, new reports have shed light on additional potential anti-inflammatory applications for this class of drugs, which confirms the role of these receptors in inflammatory and immune responses^8,9^. In this study, we blocked 5-HT_3_ receptors by using ondansetron, which significantly suppressed MAP-induced inflammation and mitigated the effects of had a significant effect of excess 5-HT exposure. Furthermore, this treatment significantly restored SERT expression and function. Collectively, these results indicate that using 5-HT_3_ receptor antagonists may have a potential role in suppressing the intestinal inflammatory response to pathogenic bacteria.

It is worth mentioning that higher SERT expression was found in the human small intestine compared with the colon^38^. Interestingly, SERT expression demonstrates remarkable variation across different regions of the human small intestine, with highest expression in the ileum, followed by the duodenum and the jejunum^38^. Low SERT expression in the colon may point out an alternate mechanism for 5-HT uptake by different monoamine transporters, including norepinephrine transporter (NET), organic cation transporter (OCT), and dopamine transporter (DAT)^38^. Whether the accumulation of 5-HT in the colonic mucosa induces expression of SERT or other monoamine transporters in the colonic epithelial cells, needs further investigation.

Since SERT is dependent on the membrane potential created by the sodium–potassium adenosine triphosphatase (Na^+^/K^+^-ATPase) to function properly, increasing the activity of this enzyme in the intestines could serve as a potential pharmacological target when SERT activity is attenuated. Despite the myriad advantages provided by Caco-2 cell culture, it does not always recapitulate the normal physiology and lineage development of the native human intestinal epithelium. Therefore, exploring SERT activity in 3-dimensional organotypic intestinal epithelium matrix will be useful to further understand 5-HT’s involvement in the intestinal pathophysiology.

In summary, our results demonstrate that TLR-2 activation by enteropathogenic infections downregulate SERT expression and function leading to reduction in 5-HT reuptake. As a result, excessive accumulation of intestinal 5-HT induces inflammation and causes tissue damage. These effects are ostensibly mediated by the p38 MAPK signaling pathway and the activation of 5-HT_3_ receptors, since inhibiting p38 MAPK pathway or blocking 5-HT_3_ receptors restored SERT activity and reduced inflammation. This indicates that TLR-2 signaling modulates the innate and the serotonergic responses, thereby directing the course of intestinal pathologies. Ultimately, regulating the intestinal serotonergic system could provide therapeutic approaches in enteropathogenic infections, which will help in understanding the gut-microbiome-brain connection.

## Materials and methods

### Clinical samples

We previously collected serum from peripheral blood samples (4.0 mL K_2_-EDTA tube) of 100 CD patients (CDAI ≥ 220 and ≤ 450) and 40 healthy control subjects acquired from Dr. Naser’s laboratory at the University of Central of Florida. MAP infection status was determined by *IS900* PCR as described earlier^39^, and then we randomly selected 40 MAP positive and 40 MAP negative CD patients for this study. Clinical serum 5-HT levels were determined by serotonin ELISA assay (Eagle Biosciences, Amherst, NH). The average age of CD patients was 45.6 ± 12.4, and the average age for healthy controls was 25 ± 5.1, respectively. The study was approved by the University of Central Florida Institutional Review Board #IRB00001138. Each subject completed and signed a written consent form before samples were collected.

### Cell culture

The in vitro part of this study was carried out in the human enterocyte-like cell line Caco-2 (ATCC HTB-37). This cell line has been described as an excellent intestinal model to study enteropathogenic infections and numerous GI disorders. Caco-2 cells (Passage #5) were cultured in ATCC-formulated Eagle's minimum essential medium supplemented with 20% fetal bovine serum and maintained at 37 °C in a humidified 5% CO_2_ incubator. Cells were grown in 12-well plates at a density of 4 × 10^4^ cells per well until confluency and differentiation have been reached.

### Bacterial infection and 5-HT treatment

Fully differentiated Caco-2 monolayers in 12-well plates were infected with 1 × 10^7^ CFU/mL of MAP, *L. monocytogenes* or *M. smegmatis* for 24 h, in the presence of exogenous 5-HT (Sigma Aldrich, St. Louis, MO) at final concentration of 0, 100, 250, 500 ng/mL. Cells were harvested and used for RNA isolation, protein extraction, SERT activity, and tissue damage assessment tests. All sets were performed in triplicates.

### Determination of SERT activity by 5-HT uptake assay

5-HT uptake measurements were performed under control conditions and following 24, 48 and 72 h of bacterial infection. The homogeneous neurotransmitter transporter uptake assay (Molecular Devices Corporation, San Jose, CA) was used to detect 5-HT uptake according to manufacturer’s protocol. The assay utilizes a fluorescent substrate that mimics the biogenic 5-HT and is taken into the cell through SERT, resulting in increased fluorescence intensity. SERT activity values were presented as percentage activity of control group without bacterial infection or 5-HT treatment.

### RNA extraction, reverse transcription and real-time PCR

Following each specific infection/treatment, total RNA was extracted and purified from Caco-2 monolayers using TRIzol isolation protocol (Abcam, Cambridge, MA). Thermal cycler (MyGene Series Peltier) was used for cDNA synthesis as described previously^40^. A total volume of 1 μL of cDNA will be mixed with 10 μL of Fast SYBR Green Mastermix (ThermoFisher, Waltham, MA), 1μL of either TNF-α, IL-6, IL-8, IL10, TLR2 and SERT (SLC6A4 gene), forward and reverse primers (ThermoFisher, Waltham, MA) and 7 μL of molecular biological grade sterile H_2_O in a 96-well microamp RT-PCR reaction plate. Controls of 18 s RNA gene oligonucleotide primers (forward primer: 5′-GTA ACC CGT TGA ACC CCA TT-3′; reverse primer: 5′-CCA TCC AAT CGG TAG TAG CG-3′) were used in order to obtain baseline CT readings. The 7500 Fast Real-Time PCR System (Applied Biosystems) was used to perform RT-PCR reaction. Relative mRNA expression levels were calculated using the equation (2^(−∆CT)^ × 1000), where ∆CT = Sample RT-PCR CT reading − 18 s CT baseline.

### Protein quantification

Following each specific infection/treatment, cell supernatants were collected, then each well of Caco-2 monolayers was incubated with 200 uL of accutase cell detachment solution (Stemcell Technologies, Cambridge, MA) for 20 min at 37 °C in a humidified 5% CO_2_ incubator. Cells were then centrifuged at 1000 rpm for 5 min and the pellets were collected and washed with PBS. Total protein was extracted from each pellet by incubating it with 200 uL of RIPA buffer (ThermoFisher, MA) for 15 min on ice, then centrifuged at 14,000 rpm for 20 min. ELISA kits specific to all cytokines/markers were measured in collected supernatant [TNF-α, IL-6, IL-8, and IL10 (ThermoFisher, Waltham, MA)] or cell lysate (SERT (MyBioSource, San Diego, CA) and TLR2 [RayBiotech, Peachtree Corners, GA)] following manufacturer’s instructions. All ELISA experiments were performed in triplicates, and the absorbance was read at 450 nm wavelength.

### Tissue damage and oxidative stress measurement

Markers of oxidative stress in fully differentiated Caco-2 monolayers including lactate dehydrogenase (LDH), glutathione (GSH), and malondialdehyde (MDA) were tested following each specific infection/treatment by using specific assay kits (ThermoFisher, Waltham, MA) per manufacturer’s protocol as we described earlier^41^.

### Treatment with TLR-2, ERK, p38MAPK and cAMP/PKA inhibitors

To confirm the role of TLR-2 activation in downregulating SERT activity following bacterial infection, we blocked TLR-2 receptors using selective human TLR-2 antagonist (Novus Biologicals, Littleton, CO) to rule out the role of other TLRs in regulating SERT function. Caco-2 monolayers were pre-incubated with 5 ug/mL of TLR2 inhibitor for 30 min prior to MAP infection and 5-HT treatment. We also assessed TLR-2 intracellular signaling pathways in depth by pre-incubating Caco-2 monolayers with selective blockers of ERK (40 uM PD98059; Cayman Chemical, Ann Arbor, MI), cAMP/PKA (1 uM KT5720; Cayman Chemical, Ann Arbor, MI) and p38 MAPK (1 uM SB220025; Sigma Aldrich, St. Louis, MO) for 30 min prior to MAP infection and 5-HT treatment (500 ng/mL).

### Ondansetron treatment

In order to reverse the effects of excess 5-HT accumulation in MAP-infected Caco-2 monolayers, we blocked 5-HT_3_ receptors with ondansetron at a concentration of 40 ng/mL for 30 min prior to MAP infection and 5-HT treatment (500 ng/mL).

### DHE and SERT fluorescence staining assay

DHE fluorescence staining was performed on Caco-2 monolayers following 24 h of MAP infection and 5-HT treatment (500 ng/mL). Caco-2 monolayers were grown on Falcon 8-well chambered cell culture slides (ThermoFisher, Waltham, MA), then washed with PBS and fixed with 4% paraformaldehyde (PFA). Monolayers were treated with 1 uM DHE stain (Sigma Aldrich, St. Louis, MO) for 25 min. Cell culture slides were examined on confocal microscope, where red staining indicates oxidative stress. To detect SERT localization, we used anti-SERT recombinant Rabbit monoclonal antibody (2 µg/mL) (ThermoFisher, Waltham, MA), labeled with Goat anti-Rabbit IgG superclonal secondary antibody conjugate (1:2000) (ThermoFisher, Waltham, MA). Caco-2 monolayers were stained for detection of SERT protein (green), nuclei is stained in blue (DAPI) and then both images were merged together. Slides were examined under Amscope IN480TC-FL-MF603 Fluorescence Microscope. Captured images were analyzed by measuring average integrated density using NIH Image J 1.39o software, which was also used to generate merged images.

### Measuring phosphorylation activity of p38 MAPK

Phospho-p38 MAPK (T180/Y182) + Total In-Cell ELISA Kit (Abcam, Cambridge, MA) was used to measure phosphorylation activity of p38 MAPK per manufacturer’s instructions. The effect of MAP infection on the ratio of p-p38 MAPK/p38 MAPK in the presence of different levels of 5-HT (0, 100, 250, 500 ng/mL) was presented with or without 1 uM of p38 MAPK inhibitor (SB220025) following 24 h of incubation.

### Statistical analysis

Statistical analysis was performed using GraphPad Prism 7.02 software. All data collected in this study were pre-tested for normal distribution using the Kolmogorov–Smirnov normality test. Significance among experiments was assessed by two-way analysis of variance (ANOVA) followed by Bonferroni correction test. For experiments performed at 3 time points over 72 h, significance was determined by repeated measures ANOVA. *P* value < 0.05 and a 95% confidence interval (CI) were used for the assessment of differences in all experiments. *P* values < 0.001 were also mentioned when achieved. Data were presented as Mean ± SD.

### Patient consent for publication

Obtained.

### Ethics approval

University of Central Florida Institutional Review Board (IRB00001138).

## Data Availability

All experiments were performed in accordance with relevant guidelines and regulations. Raw data is available upon request.
